# Structural Analysis and Design of Chionodracine-Derived Peptides Using Circular Dichroism and Molecular Dynamics Simulations

**DOI:** 10.3390/ijms21041401

**Published:** 2020-02-19

**Authors:** Stefano Borocci, Giulia Della Pelle, Francesca Ceccacci, Cristina Olivieri, Francesco Buonocore, Fernando Porcelli

**Affiliations:** 1 Department for Innovation in Biological, Agrofood and Forest Systems, University of Tuscia, 01100 Viterbo, Italy; borocci@unitus.it (S.B.); giulia.dellapelle@studenti.unitus.it (G.D.P.); fbuono@unitus.it (F.B.); 2 CNR—Institute for Biological Systems, Via Salaria, Km 29.500, 00015 Monterotondo, 00015 Rome, Italy; 3 CNR—Institute for Biological Systems, Sede Secondaria di Roma-Meccanismi di Reazione, 00185 Rome, Italy; francesca.ceccacci@cnr.it; 4 Department of Biochemistry, Molecular Biology and Biophysics, University of Minnesota, Minneapolis, MN 55455, USA; colivier@umn.edu

**Keywords:** chionodracines, antimicrobial peptides, circular dichroism, molecular dynamics, peptide-membrane interaction

## Abstract

Antimicrobial peptides have been identified as one of the alternatives to the extensive use of common antibiotics as they show a broad spectrum of activity against human pathogens. Among these is Chionodracine (*Cnd*), a host-defense peptide isolated from the Antarctic icefish *Chionodraco hamatus*, which belongs to the family of Piscidins. Previously, we demonstrated that *Cnd* and its analogs display high antimicrobial activity against ESKAPE pathogens (*Enterococcus faecium, Staphylococcus aureus, Klebsiella pneumoniae, Acinetobacter baumannii, Pseudomonas aeruginosa* and *Enterobacter* species). Herein, we investigate the interactions with lipid membranes of *Cnd* and two analogs, *Cnd-m3* and *Cnd-m3a*, showing enhanced potency. Using a combination of Circular Dichroism, fluorescence spectroscopy, and all-atom Molecular Dynamics (MD) simulations, we determined the structural basis for the different activity among these peptides. We show that all peptides are predominantly unstructured in water and fold, preferentially as α-helices, in the presence of lipid vesicles of various compositions. Through a series of MD simulations of 400 ns time scale, we show the effect of mutations on the structure and lipid interactions of *Cnd* and its analogs. By explaining the structural basis for the activity of these analogs, our findings provide structural templates to design minimalistic peptides for therapeutics.

## 1. Introduction

Antimicrobial resistance towards widely used antibiotics has become a global health crisis [1]. The diffusion of multidrug resistant (MDR) bacteria is widespread and infections caused by human pathogens often require multiple treatment, which not always effective. The United States Food and Drug Administration listed 21 qualifying pathogens that pose serious threats to public health (part 317.2 of CFR Annual Print Title 21 Food and Drugs) [2]. The search for new antimicrobial drugs to replace or integrate classical antibiotics is imperative. Many different alternatives have been proposed as potential drugs for the treatment of resistant bacterial strains such as antibodies, vaccines [3], bacteriophages [4,5], and antimicrobial peptides [6,7]. Antimicrobial peptides (AMP) represent the first line of innate immune system and are present in plants, microorganisms, and animals [8,9]. The Antimicrobial Peptide Database (APD: aps.unmc.edu/AP) comprises about 3100 entries from six kingdoms (bacteria, archaea, protists, fungi, plants, and animals) with antibacterial, antiviral, antifungal, and antiparasitic activity. Despite their different primary sequences and structures, AMPs share common properties such as cationicity, charge distribution, hydrophobicity, and amphipathicity [10,11]. Amphipathic cationic α-helical peptides have been proposed as potential antimicrobial agents due to their broad-spectrum and instantaneous antimicrobial activity [12,13,14,15,16]. However, a clear relationship between amino acid composition and antimicrobial activity still remains to be elucidated.

Antimicrobial peptides perform their function through several mechanisms such as inhibition of protein and/or DNA synthesis or inhibition of specific enzymatic pathways [17]. However, the majority of AMPs interacts with the anionic bacterial membranes and kills bacteria via cell membrane disruption [18,19]. The amphipathic character of AMP drives the interaction with the bacterial cell membrane and the subsequent disruption that kills the target cells rapidly. Two main mechanisms have been proposed: (a) pore formation and (b) carpet-like model of aggregation. The latter involves essentially four major mechanistic steps: attraction, attachment, insertion, and permeation [20,21,22,23]. The first two steps, attraction of peptide toward the cell membrane and attachment, are driven by electrostatic interactions between the positively charged AMP and the negatively charged membrane of bacteria. The two succeeding steps, insertion and permeation, are driven by the amphipathic distribution of hydrophobic and hydrophilic residues on peptide [24,25].

Chionodracine (*Cnd*) is a 22-residue antimicrobial peptide (AMP) isolated from the gills of the Antarctic fish *Chionodraco hamatus* belonging to Piscidins [26], a family of α-helical antimicrobial peptides identified in teleost fish [27]. In previous studies, we isolated *Cnd* [28] and showed that this short peptide binds lipid vesicles, disrupting the outer membrane of *E.coli* and *Psycrobater* sp. Upon membrane interactions, *Cnd* folds into a dynamic α-helix [29]. Based on our findings, we exploited *Cnd* as starting scaffold to design a series of mutants with higher positive charge to increase their antimicrobial activity and selectivity towards prokaryotic organisms. For drug design purposes, an ideal antimicrobial peptide should have maximal antimicrobial activity and minimal cytotoxicity towards eukaryotic cells. We then characterized the interactions of these peptides with model lipid vesicles and showed that they are active against MDR nosocomial bacteria strains [30,31].

In this paper, we studied more in details the structural basis for the differences in the bactericidal activity between *Cnd* and the two promising *Cnd* analogs, *Cnd-m3* and *Cnd-m3a* reported in our previous studies [30,31]. Specifically, we first determined the secondary structure of *Cnd-m3* and *Cnd-m3a* in presence of different LUVs and, afterwards, we performed all-atom MD simulations to elucidate the complex mechanism of peptide-lipid interactions and the associated conformational changes. These data could be of interest to identify the mutations useful to design new analogs with enhanced biologic activity against human bacterial pathogens.

## 2. Results

### 2.1. Peptide Design and Physico-Chemical Properties

*Cnd-m3* and *Cnd-m3a* are synthetic antimicrobial peptides designed by modifying the parent peptide *Cnd* (Table 1) [30,31] found in the gills of the Antarctic teleost fish *Chionodraco hamatus*.

**Figure 1:**
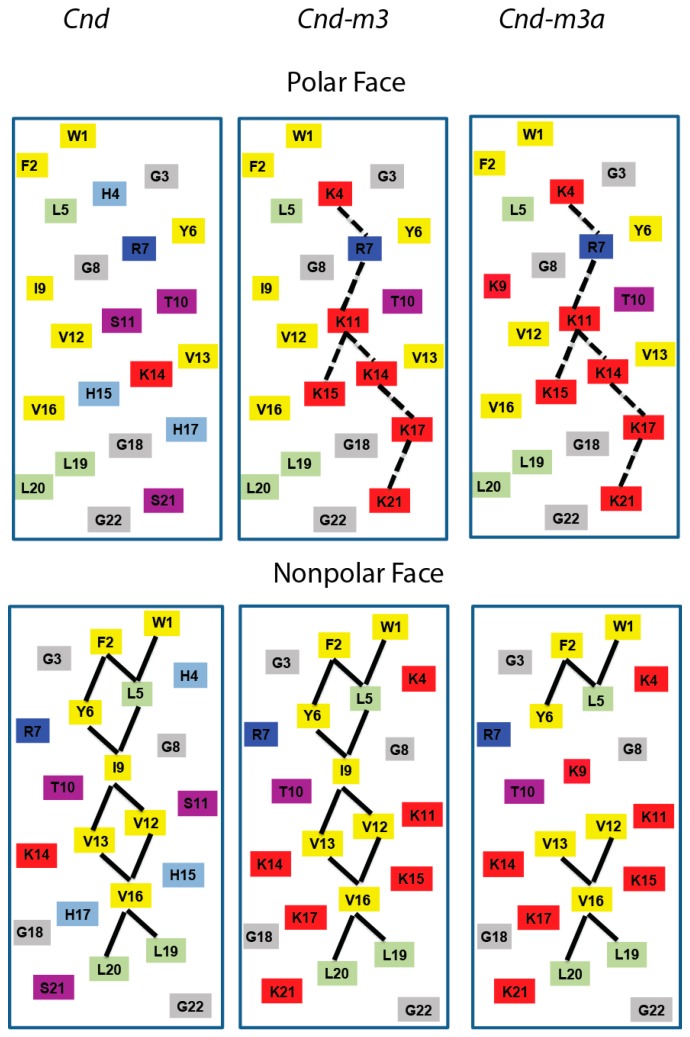
Helical net representation of *Cnd* and *Cnd*-mutants.

The number of positive charges is critical for the activity of AMP due to the interaction with the negatively charged membranes of bacteria [32]. We modified the parent peptide by introducing charged residues (i.e., Lysines) to increase its net positive charge. For *Cnd-m3*, Ser-11, Ser-22, and three Histidines in position 4, 15 and 17, respectively, were replaced by Lysines. The net positive charge increased from +2 to +7 compared to *Cnd*. For the *Cnd-m3a* mutant, Ile-9, positioned in the middle of the hydrophobic face of peptide, was replaced by Lysine, resulting in a net positive charge of +8. The helical representation of *Cnd* and its analogs is reported in Figure 1. The mutations increased the electrostatic repulsions between charged residues (*i -> i* + 3 and *i -> i* + 4) on the polar face of the putative helical structure but had no effect on the nonpolar face. *Cnd* and *Cnd-m3* displayed 11 hydrophobic interactions. The thermodynamic characterization of the interaction between peptides and vesicles made 100% of POPC and of POPC/POPG (molar ratio 70:30) was carried out and the data show that both peptides display a marked preference for lipid mixtures mimicking the prokaryotic cell membranes (POPC/POPG) [30,31]. Moreover, we showed that the antimicrobial activity of *Cnd-m3* with respect to *Cnd* increased against MDR nosocomial bacteria strains. For instance, we showed that the MIC decreased by two times for MRSA (Methicillin resistant *Staphylococcus aureus*) to 30 times for KPC (*Klebsiella pneumoniae* carbapenemase) [30]. The antimicrobial activity of *Cnd-m3a* decreased as compared to *Cnd-m3*, however, its selectivity ratio increased from 2.4 to 4.9 [31]. Moreover, in silico analysis, carried out using the Peptide Cutter tool available on EXPASY Server (https://web.expasy.org/peptide_cutter/), showed that both mutants were more resistant than *Cnd* to proteolytic degradation, especially towards the chymotrypsin-low specificity.

### 2.2. Secondary Structure Analysis by Circular Dichroism

**Figure 2:**
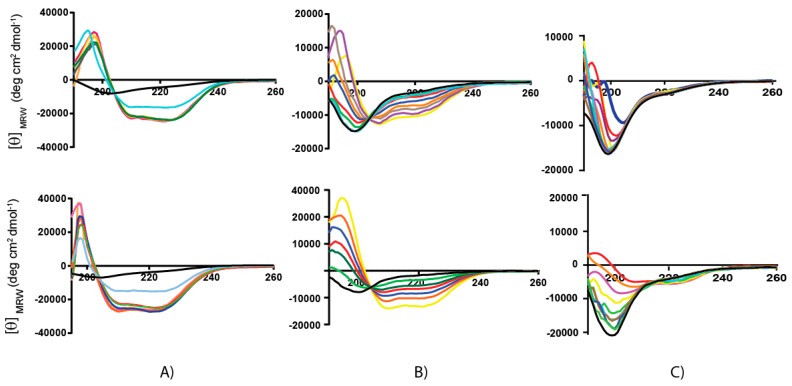
CD spectra in buffer (black line) and in the presence of increasing amount of POPC (Top line) and POPC/POPG (Bottom line) vesicles. (**A**) *Cnd*, (**B**) *Cnd-m3* and (**C**) *Cnd-m3a*. The P:L ratio ranges from 1:0 to 1:60.

The secondary structure of Chionodracine and *Cnd*-analogues was investigated by CD spectroscopy. The experiments were carried out at 25 °C in buffered LUVs, consisting of either 100% POPC or a 70:30 mixture of POPC/POPG. CD is a rapid and convenient spectroscopic technique for determining the conformational transition of proteins and peptides. We studied the conformational transitions of the three peptides upon interaction with anionic and zwitterionic membrane mimicking prokaryotic and eukaryotic environments, respectively. Typical titration curves in the presence of increasing amounts of lipid vesicles are shown in Figure 2. Increasing peptide:lipid ratios (P:L) were tested in a range between 1:0 and 1:60.

In the absence of lipid vesicles (P:L = 1:0), all the peptides were essentially unstructured, with CD spectra showing a minimum at 200 nm characteristic of a random coil structure (Black curve). In the presence of POPC vesicles, mimicking the eukaryotic cell membrane, the CD spectra of both *Cnd* and *Cnd-m3* revealed two minima at ~208 and ~222 nm, which increase with the lipid concentration, and a strong maximum at ~190 nm, typical of α-helix conformation. The rotational strength of the bands at 208 and 222 nm are often used as an index of the presence of helical structures [33,34]: for α-helical polypeptides the intensities of the two bands are almost the same. The analysis of shape of the CD spectrum as well as the ratio *θ_222nm_*/*θ_208nm_* ~ 1 confirms that *Cnd* and *Cnd-m3* fold in a canonical α-helix in presence of lipid vesicles. For the *Cnd-m3a* mutant, the CD spectra do not change significantly upon addition of lipids. In the absence of lipids, the spectra show a minimum at ~200 nm, which is typical of a random coil conformation. In presence of increasing amounts of POPC LUVs, this minimum shifts to 206 nm and a very small signal at 222 nm appears only upon addition of a large excess of LUVs (P:L ~ 1:60). The behavior of *Cnd* upon interaction with POPC/POPG (70:30) LUVs is similar to that observed in the presence of POPC vesicles, i.e., the peptide folds in a α-helix conformation at low P:L ratios. In the case of *Cnd-m3*, the value of *θ_222nm_* are higher as compared to those observed in POPC, indicating that *Cnd-m3* is more prone to adopt a helical conformation in presence of negatively charged membranes. The helicity becomes even more apparent at a P:L ratio of 1:4 and reaches the maximum at 1:30. From the analysis of CD spectra, it is apparent that in presence of POPC/POPG LUVs the value of ratio *θ_222_*/*θ_208_* can be either less than 1 or greater than 1 depending on the L/P ratio. However, the use of this ratio to distinguish between different helical conformations, such as 3_10_ or α, is not always possible and should be used with extreme caution, since the ratio depends also by the helical chain length, and aggregation to form helical bundle [33,35].

For *Cnd-m3a*, the CD spectra in presence of POPC/POPG (70:30) LUVs are similar to those in POPC, but the *θ_222nm_* values are higher. The values of *θ_222nm_* and the percentage of helicity are reported in Table 2. The molar ellipticities in POPC correspond to a helical fraction of 0.63, 0.31, and 0.12 for *Cnd, Cnd-m3*, and *Cnd-m3a*, respectively. The helical fractions (*f*_α_) in the presence of POPC/POPG were calculated according to equation 1(see Material and Methods) and resulted to be 0.64, 0.79, and 0.14, respectively. For each peptide, the structural differences detected in POPC and POPG are reported as the molar ellipticity ratio at 222 nm in POPC and POPC/POPG lipid systems:$\;\theta_{222nm}^{PC}/\theta_{222nm}^{PCPG}$ [36]. While for *Cnd* the helicities in POPC and POPC/POPG vesicles were similar, for *Cnd-m3*, the increased amphipathicity (*µH* = 0.68) favored the increase of helicity in the presence of negatively charged vesicles (i.e., POPC/POPG). The large decrease of helicity for *Cnd-m3a* with respect to *Cnd* and *Cnd-m3* was probably due to the disruption of the hydrophobic surface caused by the substitution of Ile with the positively charged Lysine in position 9.

These results agree with the partition data previously published, showing partition constants for *Cnd* and *Cnd* mutants higher in POPC/POPG than in pure POPC [30,31].

### 2.3. Steady-State Anisotropy

**Figure 3:**
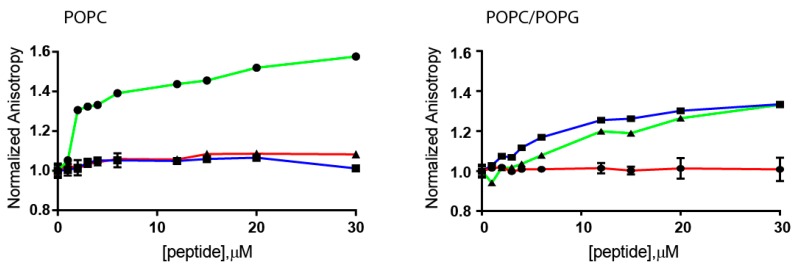
Fluorescence Anisotropy of DPH. Normalized fluorescence anisotropy of DPH in POPC and POPC/POPG lipid vesicles treated with *Cnd* (green), *Cnd-m3* (blue) and *Cnd-m3a* (red). The data are shown as mean ± s.d. from experiments performed in quadruplicate.

We also evaluated the interactions of *Cnd* and its derived peptides with lipid bilayers formed by POPC and POPC/POPG LUVs using steady-state anisotropy (*r*) of DPH (1,6-Diphenyl-1,3,5-hexatriene). DPH is a compound whose fluorescence anisotropy depends on the membrane fluidity [37]. DPH acts as a rod-like hydrophobic probe rotating in the bilayer structure and is located between the acyl chains of the lipids. Due to the presence of the lipid chains, its molecular rotation is restricted, i.e., in the presence of a high organized bilayer the rotational freedom is limited. Changes in the organization of the lipid bilayer, e.g., increasing fluidity, affect the rotational freedom of DPH and the anisotropy will change. In Figure 3, the normalized anisotropy of DPH measured at 25 °C at different concentrations of peptides is reported.

Fluorescence experiments showed that peptides interact with LUVs of different composition perturbing the hydrocarbon chain region in different ways. In the presence of POPC, only *Cnd* is able to perturb the anisotropy of DPH; while *Cnd*-mutants do not. In presence of negatively charged lipid POPC/POPG, both *Cnd* and *Cnd-m3* perturb the anisotropy of DPH while *Cnd-m3a* does not. The detected increase of the anisotropy of DPH suggests that the interaction with *Cnd* and *Cnd-m3* significantly decreased the fluidity of lipid bilayer. This latter effect could be correlated to a moderate structural organization upon interaction with the peptide. For both LUVs, *Cnd-m3a* does not induce changes in membrane fluidity. Thus, the variation of membrane fluidity is not directly correlated with the antibacterial activity. In fact, all the peptides showed antimicrobial activity but different values of DPH anisotropy. However, these differences could be related with the different mode of action of the three peptides. In fact, *Cnd* and *Cnd-m3* are membranolytic peptides [29,30] while *Cnd-m3a* can permeate through the bacteria cell membrane and directly interact with intracellular nucleic acid [31]. These results in combination with CD investigations suggest that the interaction of *Cnd* and *Cnd-m3* with LUVs is different from that of *Cnd-m3a* and such diversity is correlated with a different propensity to adopt a helical conformation.

### 2.4. Molecular Dynamics Simulation

To characterize the effects of mutations on *Cnd*-lipid interactions, we carried out atomistic MD simulations in different explicit environments such as water, TFE/water mixture, and lipid membranes composed of 100% POPC and 70:30 mixture of POPC:POPG. In the following sections, we report the results observed in of the different environments.

#### 2.4.1. Water and TFE/Water Solution

**Figure 4:**
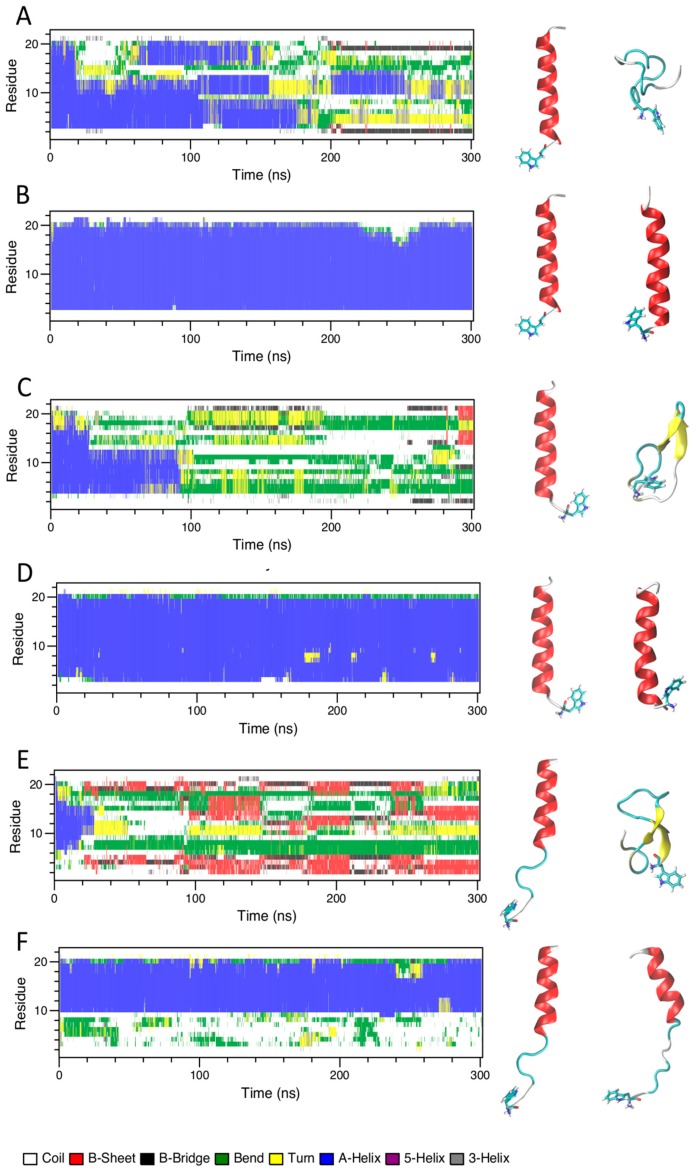
Secondary structure of *Cnd* and *Cnd* mutants in water and in TFE/water as a function of simulating time. (**A**) *Cnd* in water, (**B**) *Cnd* in TFE/water (30% *v*/*v*), (**C**) *Cnd-m3* in water, (**D**) *Cnd-m3* in. TFE/water (30% *v*/*v*), (**E**) *Cnd-m3a* in water, (**F**) *Cnd-m3a* in TFE/water (30% *v*/*v*). The structures on the right are the initial (left) and the final conformations of peptides after 300 ns of MD simulation in the corresponding environment.

Figure 4 shows the time evolution of the secondary structures for *Cnd*, *Cnd-m3*, and *Cnd-m3a* simulated in water and TFE/water (30% *v*/*v*) solutions. According to the NMR structure of *Cnd* [29] and the model of *Cnd-m3* and *Cnd-m3a* obtained by I-TASSER bioinformatics tool, *Cnd* and *Cnd-m3* were initially in α-helix conformation, while for *Cnd-m3a* the initial structure showed only ~50% of α-helix. The simulation of *Cnd* in water showed a loss of the α-helix conformation (Figure 4A) and the adoption of an unstructured conformation with the formation of short and transient α-helix involving four amino acids. Conversely, in TFE/water mixture, *Cnd* conserved the α-helix throughout the entire simulation (Figure 4B) and the structure is similar to the NMR structure obtained in DPC micelles, with a backbone RMSD of 0.12 nm. In water, *Cnd-m3* is more flexible than *Cnd* and remains essentially unstructured during simulation with the formation of two short antiparallel β-sheets in the C-terminal regions of peptide in the last 10 ns (Figure 4C). Similar to *Cnd*, the simulation of *Cnd-m3* in TFE/water shows the presence of a stable α-helix that is substantially preserved throughout the entire simulation (Figure 4D). The initial structure of *Cnd-m3a* contains 10 residues of the C-terminus in an α-helical conformation; whereas the N-terminus is unstructured. This conformation is persistent both in water and TFE/water (Figure 4E,F).

Overall, all peptides showed that in TFE/water (30% *v*/*v*) their initial α-helix structures are stabilized and preserved during the MD simulation.

#### 2.4.2. Peptides with Lipid Bilayers

**Figure 5:**
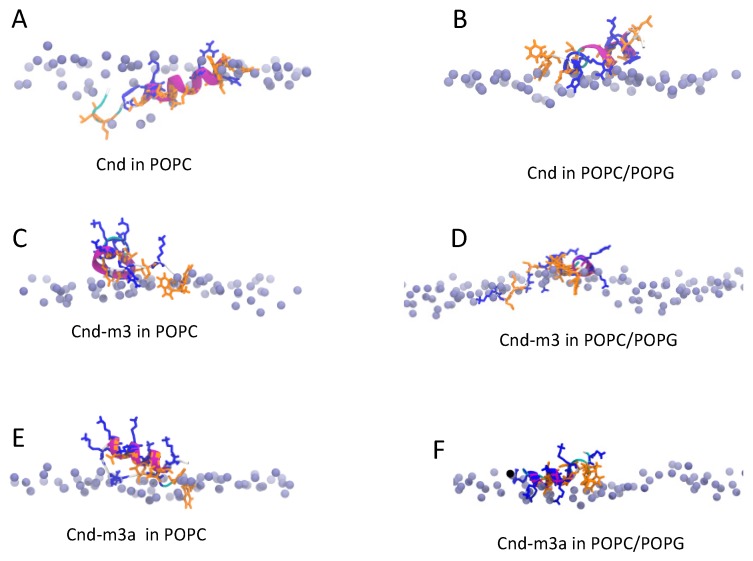
Snapshot MD simulation at 400 ns of (**A**) *Cnd* in POPC, (**B**) *Cnd* in POPC/POPG, (**C**) *Cnd-m3* in POPC, (**D**) *Cnd-m3* in POPC/POPG, (**E**) *Cnd-m3a* in POPC and (**F**) *Cnd-m3a* in POPC/POPG. The phosphate of the upper leaflet of bilayer is represented as violet dots. The residue of lysine, arginine and histidine are represented in blue stick whereas the hydrophobic amino acids are represented as orange stick. The water and counterions are omitted for clarity.

The folding of peptide at water/membrane surface is a process with a time scale in the range of milliseconds to seconds. Therefore, to reduce computational cost, we studied the interactions of *Cnd* and its mutants with lipid bilayer starting from their equilibrated conformations in TFE/water. The α-helical of peptides were taken parallel to lipid surface with a distance of 0.5 nm between the center of mass of peptide and the phosphate atoms of lipids. *Cnd* placed close to the surface of bilayer of POPC and POPC/POPG conserves a helicity of 55% ± 3% and 50% ± 5%, respectively (Figure S1). Upon interaction with the POPC head groups *Cnd* is adsorbed on the membrane/water interface, pointing its hydrophobic residues toward the hydrophobic region of bilayer; whereas the charged Lys-14 and Arg-7 and the Histidine residues (His-4, His-15, His-17) are oriented towards the bulk water phase (Figure 5A). In POPC/POPG bilayer, *Cnd* shows a similar orientation of the amphipathic helix as observed for the simulation in POPC. However, the peptide snorkels toward the water/membrane interface due to the presence of arginine and histidine residues that interact with the phosphate groups (Figure 5B).

**Figure 6:**
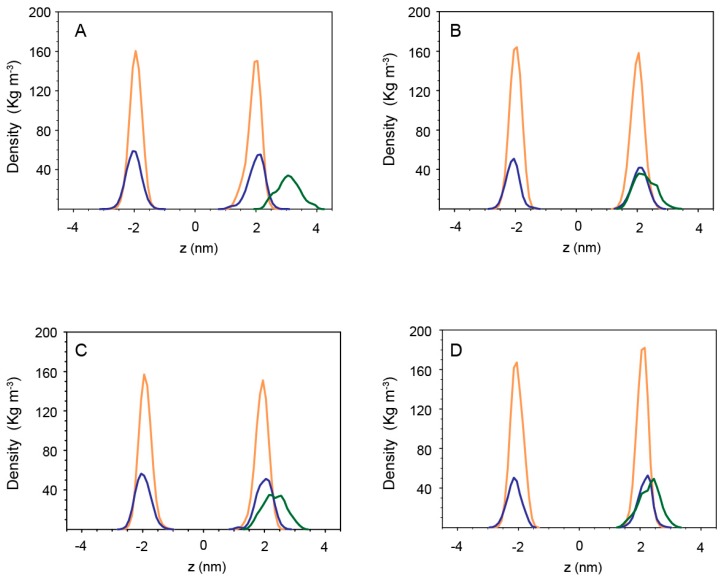
Mass density profiles across lipid bilayer of lipid phosphate (orange), nitrogen atoms of choline (blue) and lysine residues (green) of (**A**) *Cnd-m3* in POPC, (**B**) *Cnd-m3* in POPC/POPG, (**C**) *Cnd-m3a* in POPC, (**D**) *Cnd-m3a* in POPC/POPG. The mass density is calculated with respect to the lipid bilayer center (z = 0).

The presence of a high number of lysines, seven in *Cnd-m3* and eight in *Cnd-m3a*, plays a crucial role on the structure of peptides and on their interactions with lipids. *Cnd-m3* and *Cnd-m3a*, located on the surface of POPC bilayer, conserved only a fraction of the initial helicity (25% ± 4% for *Cnd-m3* and 28% ± 3% for *Cnd-m3a*) in the middle region of the peptide. (Figure S1) Lysines and arginines are oriented toward the water phase and the interaction of *Cnd-m3* and *Cnd-m3a* with lipid hydrocarbon chains is due mainly to their hydrophobic residues. In presence of POPC/POPG, the helicity of *Cnd-m3* and *Cnd-m3a* is similar to the simulations in POPC. However, the interactions between lysines and arginines with the phosphate groups of lipid are more persistent (Figure 5C–F). Figure 6 shows the mass density distribution of phosphate and nitrogen atoms of lipids (POPC and POPC/POPG) and the lysine residues of *Cnd-m3* and *Cnd-m3a* calculated, in the final 100 ns of trajectory with respect to the center of the lipid bilayer.

The analysis of the mass density shows that in the interaction with POPC bilayer the l lysine residues of *Cnd-m3* and *Cnd-m3a* are oriented toward the water phase far from the phosphate groups (Figure 6A,C). On the contrary, in the presence of negative charged lipid bilayers (POPC/POPG, Figure 6B,D), the density of lysine residues overlaps with that of the phosphate groups, indicating significant interactions between the charged groups of peptides and lipids. When inserted perpendicularly to the lipid bilayer, the peptides show a more defined conformation with respect to the peptides parallel to the surface of bilayer. *Cnd* embedded into POPC and POPC/POPG bilayer preserves a stable α-helical conformation during the entire simulation (400 ns) with a helicity of 82% ±2% and 81% ± 2% (18 residues), respectively. In POPC and POPC/POPG bilayers, *Cnd* adopts a more helical structure with respect to both *Cnd-m3* and *Cnd-m3a* mutants. The helical content of *Cnd-m3* in POPC is 45% ±2% (10 residues) and increases to 65% ± 3% (14 residues) in the presence of POPC/POPG.

**Figure 7:**
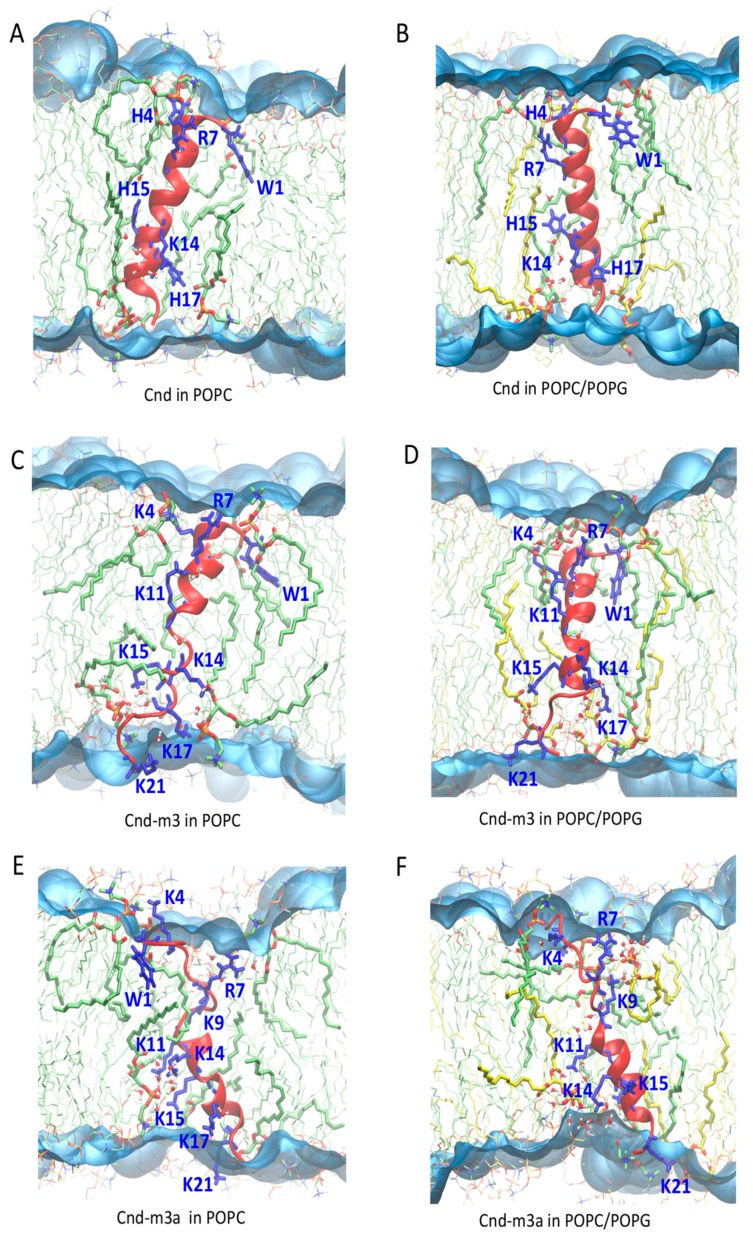
Snapshot at the end (400 ns) of MD simulations of *Cnd*, *Cnd-m3* and *Cnd-m3a* embedded in lipid bilayer: (**A**) *Cnd* in POPC, (**B**) *Cnd* in POPC/POPG, (**C**) *Cnd-m3* in POPC, (**D**) *Cnd-m3* in POPC/POPG, (**E**) *Cnd-m3a* in POPC, (**F**) *Cnd-m3a* in POPC/POPG. The secondary structure of peptides is represented in cartoon and colored in red, lysine arginine, histidine and tryptophan are represented in blue sticks. The lipid molecules are represented in thin sticks and colored according to their atom types (red for oxygen, blue for nitrogen, orange for phosphate atoms, green for carbon atoms of POPC, yellow for carbon atoms of POPG). The lipid molecules within 3.5 Å from the peptide atoms are represented in sticks. Water molecules in the hydrophobic region of lipid are represented in ball-sticks. The water surface is generated using the VMD Surf representation.

*Cnd-m3a*, embedded in POPC and POPC/POPG bilayer, shows a stable α-helix in the C-terminus with a helical content of 47% ± 3% in POPC and 44% ± 3% in POPC/POPG. Figure 7 shows the position of the peptides with respect to the lipid bilayers (POPC and POPC/POPG) at the end of each MD simulation. For *Cnd-m3* and *Cnd-m3a*, the insertion of lysine residues into the hydrophobic core of lipid bilayer induces a perturbation of the lipid aggregate. In fact, the hydrophobic side chains of lysines, positively charged, orient toward the negative charged phosphate group of lipids (snorkel effect [38]). As shown in Figure 7, the lipid molecules close to the peptide change partially the orientation to have favorable interactions. The presence of lysine residues in the hydrophobic regions of bilayer determines also the penetration of water molecules (5–7 molecules) that interact with the charged amino acid by hydrogen bonds. The presence of water molecules in the hydrophobic core, together to the presence of charged lysines, contributes to destabilize the lipid bilayer, which shows a reduced thickness in the region near the peptides.

**Figure 8:**
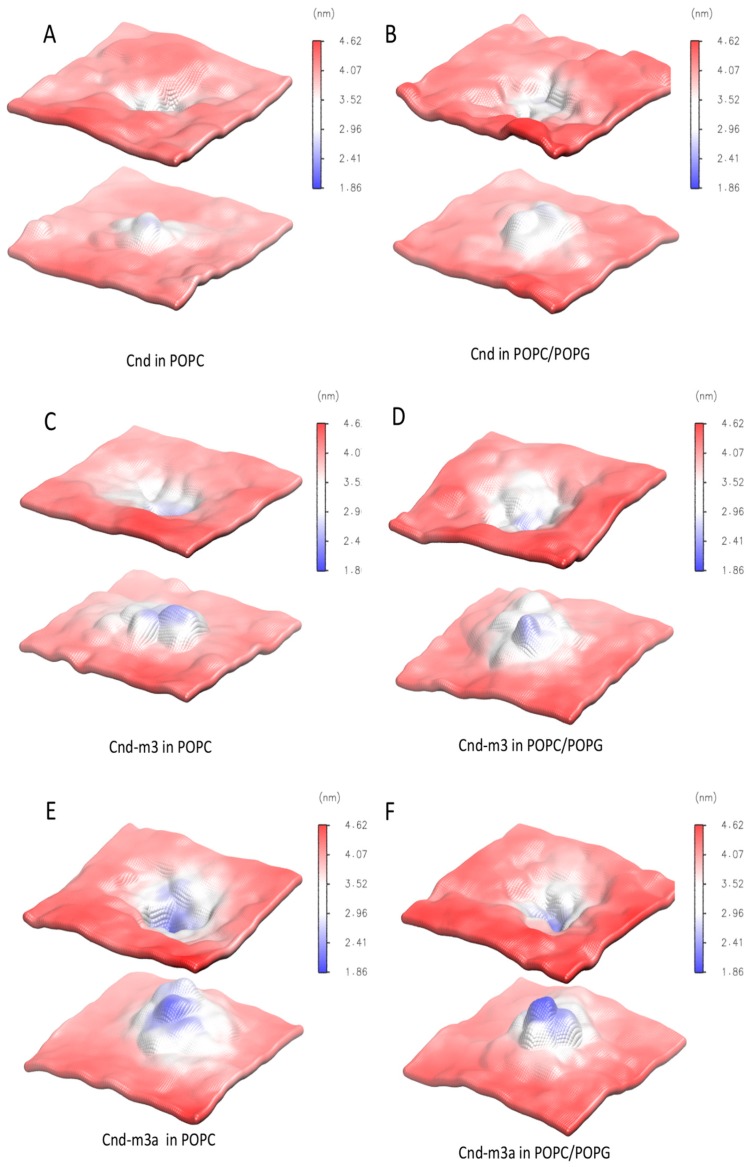
Time average of the last 100 ns of simulations of POPC and POPC/POPG local lipid bilayer thickness in presence of antimicrobial peptides embedded in to lipid bilayer: (**A**) *Cnd* in POPC, (**B**) *Cnd* in POPC/POPG, (**C**) *Cnd-m3* in POPC, (**D**) *Cnd-m3* in POPC/POPG, (**E**) *Cnd-m3a* in POPC, (**F**) *Cnd-m3a* in POPC/POPG. The thickness increases as a blue-white-red color gradient.

The destabilizing effects of *Cnd*, *Cnd-m3* and *Cnd-m3a* on the structure of lipid bilayer can be evidenced by analyzing the change of thickness of lipid bilayer during the last 100 ns of each MD simulations. Figure 8 shows the 3D density maps of time averaged thickness of POPC and POPC/POPG in presence of *Cnd*, *Cnd-m3* and *Cnd-m3a* embedded in lipid bilayer. The blue color corresponds to the region of bilayer with low values of thickness and the red color to the region with high thickness. The peptides embedded in bilayer are localized in the region with the lowest thickness.

As shown in Figure 8 the thickness of lipid bilayer was perturbed around the antimicrobial peptides. The largest structural deformation of lipid bilayer, both in POPC and POPC/POPG, was observed in the case of *Cnd-m3a* with the average bilayer thickness in vicinity of the peptide decreased approximately up to 1.9 nm. In the case of *Cnd-m3* the averaged thickness of lipid bilayer close to peptide was approximately of 2.5 nm. *Cnd* induces a perturbation of thickness smaller with respect to *Cnd-m3a* and *Cnd-m3* with the average bilayer thickness in vicinity of the peptide approximately of 2.9 nm.

## 3. Discussion

Alternative approaches are needed to neutralize antibiotic resistant diseases. Host defense peptides such as AMPs offer a good starting model to obtain more active antimicrobial agents. More than 3000 AMPs are reported in public database [39] giving a good source of information to delineate strategies for the AMP design. Here, we selected the two *Cnd* analogues, which showed a potent antimicrobial activity against multidrug resistant bacteria and investigated their interaction with model membranes in order to gain insight on such interactions that could be useful for the rational design of novel antimicrobial agents [30,31]. These analogues were designed considering the number of positive charges, the distribution of hydrophobic residues and the location of specificity determinants. According to Hodges and co-workers specificity determinants are positively charged residues in the middle of the non-polar face of the hypothetical amphipathic helix (Figure 1) [40]. The role of these residues is to increase the selectivity between prokaryotic and eukaryotic membranes. Moreover, it has been established that high hydrophobicity on the non-polar face correlates with peptide self-aggregation to form dimers or oligomers and with a decrease in the antimicrobial activity [41]. Selective toxicity, killing bacterial pathogens without damaging eukaryotic cells, is crucial for the development of AMP. Recently, we demonstrated the increase in selectivity of *Cnd* analogs as measured by the selectivity ratios, defined by the ratio between the partition constants measured in POPC/POPG and POPC. Higher values indicate the preference of peptide to partition into anionic vesicles, mimicking the bacterial cell membrane and we found values of 1.43, 2.39 and 4.90 for *Cnd*, *Cnd-m3* and *Cnd-m3a*, respectively [30,31]. We used CD spectroscopy and all atoms MD simulations to elucidate peptide conformational changes the underlay the complex mechanism of peptide-lipid interactions.

CD spectroscopy experiments revealed a difference in the secondary structure adopted by peptides in presence of various membrane mimicking environments. All peptides adopted a random coil conformation in phosphate buffer and undergo a transition to a more ordered conformation in the presence of anionic and zwitterionic LUVs. However, *Cnd* and *Cnd-m3* fold in an α-helical conformation, both in the presence of POPC and POPC/POPG LUVs, whereas *Cnd-m3a* seems to retain its initial random coil conformation in the presence of POPC LUVs and shows a very modest helical content in POPC/POPG LUVs. The percentage of α-helix is much higher in presence of POPC/POPG compared to POPC for *Cnd-m3*, the analog with higher antimicrobial activity, with a *θ_222nm_* molar ellipticity ratio of 0.40 (Table 2). For *Cnd* and *Cnd-m3a*, the ratio was ~1 indicating the same percentage of α-helix in both anionic and zwitterionic membranes. Interestingly, *Cnd-m3a* adopts a α-helix conformation, although to a lesser extent ~14% with respect to *Cnd* and *Cnd-m3*. This indicates that the introduction of the Lysine in position 9 in the middle of the non-polar face interfere with the ability to adopt and α-helical structure. Despite the different conformation adopted by *Cnd-m3a* and its different behavior with respect to the other two peptides in the presence of POPC and POPC/POPG vesicles, its antimicrobial activity is preserved. This is in accord with previous studies, which evidenced a different mode of action of the three peptides. In fact, while *Cnd* and *Cnd-m3* have been described as membranolytic peptides, adopting an α-helical conformation to trigger their activity [30], we demonstrated that *Cnd-m3a* is able to traverse inner and outer membrane of *Psychrobacter sp.* targeting intracellular nuclei acids [31]. To get further insights into how primary structure can drive conformations, we carried out molecular dynamics simulations in different environments. MD simulations in presence of POPC and POPC/POPG bilayer agree with the experimental CD results and revealed the atomistic interactions between peptides and membranes. *Cnd* shows a higher content of helical structure with respect to the analogs in presence of both lipid bilayer of POPC and POPC/POPG. *Cnd-m3* shows an increase of helical content moving from POPC to POPC/POPG, whereas *Cnd-m3a* does not show a significant change in the helical content in the presence of anionic component POPG in bilayer. The MD simulation validates that the substitution of Ile-9 with a lysine in *Cnd-m3* with a lysine destabilizes the α-helix conformation in the N-terminal region of *Cnd-m3a*. MD simulations of the peptides absorbed on the surface of POPC bilayers emphasize that the peptides interact with the lipidic surface mainly through hydrophobic contacts. The presence of lysine residues helps to anchor the *Cnd-m3* and *Cnd-m3a* to the surface of POPC/POPG bilayer through the formation of hydrogen bonds and ion-pairs with the phosphate group of lipids (Figure 4). *Cnd*, *Cnd-m3*, and *Cnd-m3a* embedded into bilayer perturb the organization of the lipids in the aggregates, especially for *Cnd-m3* and *Cnd-m3a* due to the presence of lysine residues. The lysine residues in the hydrophobic core of lipid bilayer orient the positively charged head of side chains towards the surface (snorkeling effect) to obtain an energetically favorable interaction with the head groups of lipids. *Cnd-m3* and *Cnd-m3a* induce a reorganization of lipid components close to peptides with a sensible reduction of the thickness of bilayer.

## 4. Materials and Methods

### 4.1. Materials

All lipids were purchased from Avanti polar Lipids (Alabaster, AL, USA). All peptides were purchased from CASLO ApS, c/o Scion Technical University of Denmark, with a purity >98%. Peptide concentrations were measured before each sample preparation by UV light absorption at 280 nm. Phosphate buffer pH 7.4 was used to diminish the UV absorbance in CD experiments.

### 4.2. Preparation of Lipid Vesicles

LUVs (Large Unilamellar Vesicles) were prepared as previously reported [29]. Lipids were dissolved in chloroform/methanol 9:1. The solvent was then removed by rotary evaporation and then overnight under high vacuum. The lipid film was then hydrated by adding 1 mL of buffer (20 mM phosphate buffer at pH 7.4 with 150 mM NaCl and 0.8 mM EDTA) and then subjected to 5 freeze-thaw cycles and vortexed. The multilamellar vesicles (MLVS) formed were extruded through a polycarbonate membrane with pore size of 100 nm using a mini extruder (Avanti Polar). The obtained LUVs were composed of 100% POPC (1-palmitoyl-2-oleoyl-*sn*-glycero-3-phosphocholine) and of a 70/30 (*w*/*w*) combination of POPC/POPG (1-palmitoyl-2-oleoyl-*sn*-glycero-3-phosphoglycerol). The final concentration of the lipid was 10 mM.

### 4.3. Circular Dichroism Spectroscopy

CD spectra were recorded in the 190–260 nm spectral range at 298 K on a J 715 JASCO spectropolarimeter equipped with a Peltier device for temperature control (0.5-cm path length quartz cuvette). Secondary structures of the three peptides were determined in phosphate buffer (PB) 0.01 M and EDTA 0.08 M at pH 7.4 in the absence and presence of LUVs of different composition. Peptides were prepared as 1.0 mM stock solution. A stock solution of LUVs (10 mM) was used. For LUVs interaction studies, solutions of 30 µM of *Cnd* and Chionodracine derived peptides were titrated with LUVs of different composition (100% POPC, 70:30 POPC:POPG) with a peptide/lipid ratio ranging from 1:0 to 1:60. Contribution from buffer and LUVs were removed by subtracting their spectra in the absence of peptides. The reported CD spectra are the average of 16 scans with a scanning speed of 20 nm/min, a response time of 8 s, a bandwidth of 1.0 nm and a step size of 0.1 nm. The obtained data in millidegrees (*θ*) were converted to *mean molar ellipticities* (deg cm^2^ dmol^−1^). The fractional helical content *f_α_* has been estimated using the formula [42]:

(1) $$f_{\alpha }=\frac{\theta -\theta_{RC}}{\theta_{H}-\theta_{RC}}$$

where *θ* is the observed ellipticity at 222nm and *θ_RC_* and *θ_H_* are the limiting values for a completely random coil and α-helical conformation, respectively. At the temperature of 25 °C, according to the Luo and Baldwin formula [43], the limiting values of ellipticities are $\theta_{H}=\;-39,375$ and $\theta_{RC}=\;-360$.

### 4.4. Steady-State Anisotropy Measurements

Lipids were dissolved in chloroform/methanol 9:1 and mixed with stock solution of DPH 1,6-Diphenyl-1,3,5-hexatriene. The molar ratio DPH/Lipids was 1:1000 and the LUVs were extruded as previously described. The anisotropy measurements were performed using a Perkin Elmer LS55. The temperature was set at 25 °C using a thermostatic cell holder controlled by an external bath circulator. The samples were excited at 380 nm and the emission was detected at 420 nm with an emission and absorption bandwidth of 5 nm [44]. Fluorescence anisotropy value, *r*, were obtained according to the equation:

(2) $$r=\frac{I_{VV}-G^{*}I_{VH}}{I_{VV}+2G^{*}I_{VH}}$$

where *I_VV_* is the fluorescence measured with the excitation and emission polarizers oriented in the vertical position and *I_VH_* is the fluorescence intensity measured with the excitation polarizer oriented vertically and the emission oriented in horizontal position. G* is the correction grating factor, defined as the ratio between the vertically and horizontally polarized emission components upon excitation with a horizontally polarized light [45]. DPH acts as a rod-like hydrophobic probe rotating in the bilayer structure and located between the acyl chains of the lipids [46]. Changes in anisotropy values of DPH were used to evaluate membrane fluidity and peptide interaction with LUVs.

### 4.5. Computational Methods

#### 4.5.1. Preparation of MD Simulations

The initial structure of *Cnd* was taken from the PDB structure resolved by NMR spectroscopy as described previously [29]. The three-dimensional structures of *Cnd-m3* and *Cnd-m3a* has not been resolved yet by NMR spectroscopy. Therefore, we used the fold recognition algorithm implemented in I-TASSER (iterative threading assembly refinement algorithm) [47,48] to predict a 3D model of *Cnd-m3* and *Cnd-m3a* starting from the amino acid sequence of target peptides. The best-scored model structures obtained by the I-TASSER prediction of *Cnd-m3* and *Cnd-m3a* were used.

Titratable residues of peptides were modelled according to their protonation state in water at pH = 7.

The initial configuration of POPC and POPC/POPG (70:30) lipid bilayers was built by using PACKMOL program [49]. Each bilayer system was built with 128 lipids (64 per leaflet) and about 35 water molecules per lipid to have a fully hydrated bilayer. The mixed bilayer of POPC/POPG (70:30) was built by using 92 molecules of POPC and 36 molecules of POPG symmetrically distributed between the two bilayer leaflets. The lipid bilayers were equilibrated for 200 ns before the addition of peptides.

For each peptide six different simulation were performed:

*(1)*  *in water:* the peptides were immersed in a cubic box (5.0 nm) and solvated with at list 4000 water molecules;
*(2)*  *in a mixture of TFE*/*water (30% v*/*v):* the peptides were immersed in a cubic box (5.0 nm) and solvated with about 270 molecules of TFE and 2650 water molecules;
*(3)*  *with the peptide absorbed on lipid bilayer surface of POPC:* the peptide was positioned above the pre-equilibrated bilayer surface of POPC with the helical axis parallel to the lipid surface with a distance of 0.5 nm between the center of mass of peptide and the phosphate atoms of lipids. A rectangular box of 6.5 × 6.5 × 8.0 nm^3^ was used with at list 5140 water molecules;
*(4)*  *with the peptide embedded in POPC lipid bilayer:* the peptide was embedded in a pre-equilibrated lipid bilayer of POPC with the helical axis parallel to the normal of lipid bilayer in a rectangular box of 6.5 × 6.5 × 8.0 nm^3^ with at list 5140 water molecules;
*(5)*  *with the peptide absorbed on lipid bilayer surface of POPC:POPG:* the peptide was positioned above the pre-equilibrated bilayer surface of POPC:POPG with the helical axis parallel to the lipid surface and a distance of 0.5 nm between the center of mass of peptide and the phosphate atoms of lipids. A rectangular box of 6.2 × 6.2 × 8.0 nm^3^ was used with at list 4300 water molecule;
*(6)*  *with the peptide embedded in POPC*/*POPG lipid bilayer:* the peptide was embedded in a pre-equilibrated lipid bilayer of POPC/POPG (70:30) with the helical axis parallel to the normal of lipid bilayer in a rectangular box of 6.2 × 6.2 × 8.0 nm^3^ with at list 4300 water molecules.

For the simulation in water and in the mixture of TFE/water the NMR resolved structure of *Cnd* and the I-TASSER model of *Cnd-m3* and *Cnd-m3a* was used. The structure of peptide obtained at the end of the MD simulation in TFE/water was used for the simulations in presence of lipid bilayer of POPC and POPC/POPG. The peptides were immersed in the hydrophobic region of lipid bilayer using the embedding protocol of M. Javanainen [50].

In all systems the appropriate number of chloride ions and sodium ions was used to neutralize the charges of the peptides and POPG molecules.

#### 4.5.2. The MD Systems Setup

All MD simulations were carried out with GROMACS v.5.1.5 package [51]. The GROMOS G54a7 force field [52] was used to model the peptides molecules and ions in conjunction with the GROMOS-CKP [53,54,55] for the lipids. The phosphatidylglycerol component of the mixed lipid bilayer was a racemic mixture of 1-palmitoyl-2-oleoyl-*sn*-glycero-3-phospho-l-(1-glycerol) (L-POPG) and 1-palmitoyl-2-oleoyl-*sn*-glycero-3-phospho-d-(1-glycerol) (D-POPG). Water was modelled with the SPC model [56] and the force field proposed by Fioroni et al. [57] was used to describe TFE.

Lennard-Jones and electrostatic interactions were calculated using a cut-off of 1.2 nm and the long-range electrostatic interactions were accounted by using the particle mesh Ewald method (PME) [58].

All bonds were constrained using the P-LINCS algorithm [59,60] whereas the geometry of water molecules was fixed with the SETTLE algorithm [61]. The simulations were carried out with a time step of 2 fs.

The molecular systems were energy minimized and equilibrated as follows.

The systems were warmed up with five consecutive MD in the NVT ensemble from 100 K to 298 K in 500 ps with weak positional restraints on heavy atoms of peptides and lipids. Subsequently, 300 ns of unrestrained MD simulation in the NPT ensemble were performed for the peptides in water and in TFE/water whereas 400 ns of unrestrained MD (NPT) were performed for the peptides in presence of lipid bilayer. For each system two simulations with different random initial velocities were performed. The temperature of peptide, lipids, water and ions was kept constant separately at 298 K using the velocity rescale method [62] with a time constant of 0.1 ps. The pressure was controlled by using the Berendsen barostat [63] (P = 1 bar, τ_p_ = 1.0 ps). The pressure coupling was isotropic in the case of the simulations in water and in the mixture of TFE/water (30% *v*/*v*). In the simulation with lipid bilayer the pressure coupling was applied semi-isotropic: the z and the xy dimensions were allowed to vary independently.

Periodic boundary conditions were applied in all three dimensions.

A list of the simulations performed and details about the composition of the simulated systems are reported in Table S1.

#### 4.5.3. The MD Analyses

The trajectories obtained by MD simulation were analysed with the GROMACS analysis tools, VMD 1.9.3 [64] and in-house scripts. The analysis of trajectories was performed on the last 100 ns of the simulations.

Analysis of the secondary structure was performed with gmx do_dssp tool of GROMACS using the program DSSP [65]. The time averaged local thickness of lipid bilayer was calculated using the g_lomepro tool considering the distance of phosphate atoms of lipids in opposite membrane leaflets [66].

## 5. Conclusions

In conclusion, our results highlight the interaction between *Cnd* and its analogues with anionic and zwitterionic membranes. Our studies showed that peptides are unstructured in water and fold, preferentially in α-helical structure, in presence of lipid vesicles of different compositions. Our computational studies confirmed the experimental results through a series of MD simulations of 400 ns time scale and showed at atomic resolution the effect of the mutations on the on the structure of peptides and their interaction with lipid membranes. While *Cnd* adopts a helical conformation both in POPC and POPC/POPG vesicles at low L:P ratio, *Cnd-m3* shows a different behavior with the two types of bilayer, being more prone to adopt an α-helical in the presence of negatively charged vesicles. This correlates with previous partition studies evidencing a preference of *Cnd-m3* toward POPC/POPG vesicles. The nutation of Ile-9 with a lysine in *Cnd-m3*, in the middle of the hydrophobic face, destabilizes the α-helix at the N-terminus and brings to a peptide, *Cnd-m3a* with completely different features. Overall this study lays the groundwork for the design of small peptides that can be used as an alternative to common antibiotics to reduce the risk of antibiotic resistance.

## Figures and Tables

**Table 1:** Sequence and physicochemical characteristics of chionodracines.

Peptide	Sequence	Net Charge	pI	(μH)
***Cnd***	**W**FGHLYRGITSVVKHVHGLLSG	+2	9.99	0.574
***Cnd-m3***	**W**FG**K**LYRGIT**K**VVK**K**V**K**GLL**K**G	+7	10.58	0.684
***Cnd-m3a***	**W**FG**K**LYRG**K**T**K**VVK**K**V**K**GLL**K**G	+8	10.82	0.564

**Table 2:** Helicity in presence of different LUVs environment calculated according to the formula (1) derived by Luo and Baldwin.

	POPC		POPC/POPG (70:30)		POPC vs. POPC/POPG
	*θ* *_222nm_*	*% helicity*	*θ* *_222nm_*	*% helicity*	$\theta_{222nm}^{PC}/\theta_{222nm}^{PCPG}$
Cnd	−24,900	62.8	−25,500	64.4	0.98
Cnd-m3	−12,300	30.6	−31,000	78.5	0.40
Cnd-m3a	−5000	11.9	−5100	14.0	0.98

## Supplementary Materials

Supplementary materials can be found at https://www.mdpi.com/1422-0067/21/4/1401/s1.

## Abbreviations

AMP	Antimicrobial peptide
CD	Circular dichroism
*Cnd*	Chionodracine
DPH	1,6-Diphenyl-1,3,5-hexatriene
EDTA	Ethylenediaminetetraacetic acid
LUV	Large Unilamellar Vesicles
MD	Molecular Dynamics
MDR	Multidrug resistant
MIC	Minimum inhibitory concentration
MLVS	Multilamellar vesicles
OD	Optical Density
PB	Phosphate buffer
POPC	1-palmitoyl-2-oleoyl-*sn*-glycero-3-phosphocholine
POPG	1-palmitoyl-2-oleoyl-*sn*-glycero-3-(1′-rac-glycerol) sodium salt
TFE	2,2,2-Trifluoroethanol
