# Supplementary material for: Structural Analysis and Design of Chionodracine-Derived Peptides Using Circular Dichroism and Molecular Dynamics Simulations
Source: Int J Mol Sci. 2020 Feb 19;21(4):1401. doi: 10.3390/ijms21041401 (PMC7073106; doi:10.3390/ijms21041401)
Supplement: Supplementary file 1 [file ijms-21-01401-s001.pdf]

International Journal of
Molecular Sciences

MDPI

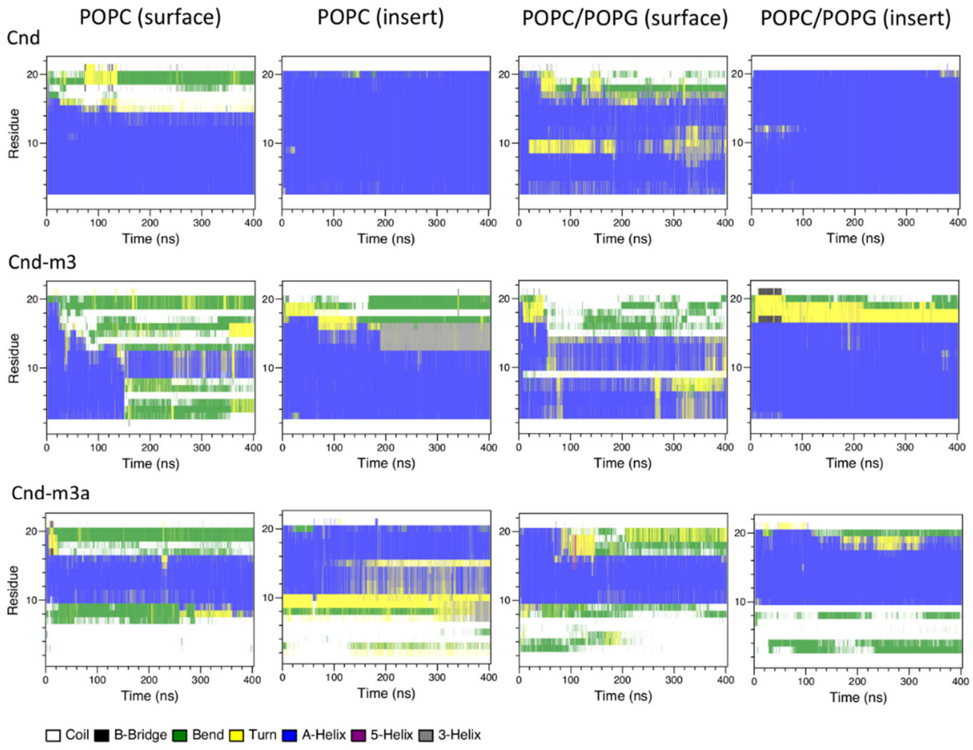

Figure S1. Secondary structure of *Cnd* and *Cnd* mutants in different membrane mimicking systems.

Table S1. Simulation Systems.

Peptide	System	Number of Molecules			Ions	Time (ns)
		Lipids	Water	TFE		
Cnd	water		4008		2 Cl^-	300
Cnd-m3	water		4003		7 Cl^-	300
Cnd-m3a	water		4004		8 Cl^-	300
Cnd	TFE/water		2650	272	2 Cl^-	300
Cnd-m3	TFE/water		2508	260	7 Cl^-	300
Cnd-m3a	TFE/water		2489	256	8 Cl^-	300
Cnd	POPC[a]	128	5141		2 Cl^-	400
Cnd	POPC[b]	128	5383		2 Cl^-	400
Cnd-m3	POPC[a]	128	5125		7 Cl^-	400
Cnd-m3	POPC[b]	128	5378		7 Cl^-	400
Cnd-m3a	POPC[a]	128	5089		8 Cl^-	400
Cnd-m3a	POPC[b]	128	5377		8 Cl^-	400
Cnd	POPC/POPG[a]	92 PC, 36PG	4317		2 Cl^-, 36Na^+	400
Cnd	POPC/POPG[b]	92 PC, 36PG	5383		2 Cl^-, 36Na^+	400
Cnd-m3	POPC/POPG[a]	92 PC, 36PG	4307		7 Cl^-, 36Na^+	400
Cnd-m3	POPC/POPG[b]	92 PC, 36PG	5218		7 Cl^-, 36Na^+	400
Cnd-m3a	POPC/POPG[a]	92 PC, 36PG	4150		8 Cl^-, 36Na^+	400

Cnd-m3a	POPC/POPG[b]	92 PC, 36PG	5217	8 Cl^-, 36Na^+	400

[a] Peptide absorbed on the bilayer surface. [b] Peptide embedded in to lipid bilayer.
